# Enhanced surveillance for Rift Valley Fever in livestock during El Niño rains and threat of RVF outbreak, Kenya, 2015-2016

**DOI:** 10.1371/journal.pntd.0006353

**Published:** 2018-04-26

**Authors:** Harry Oyas, Lindsey Holmstrom, Naomi P. Kemunto, Matthew Muturi, Athman Mwatondo, Eric Osoro, Austine Bitek, Bernard Bett, Jane W. Githinji, Samuel M. Thumbi, Marc-Alain Widdowson, Peninah M. Munyua, M. Kariuki Njenga

**Affiliations:** 1 Veterinary Epidemiology and Economics Unit, Kenya Ministry of Agriculture, livestock and Fisheries, Nairobi, Kenya; 2 College of Veterinary Medicine, Kansas State University, Manhattan, Kansas, United States of America; 3 Washington State University Global Health Program-Kenya, Washington State University, Nairobi, Kenya; 4 Kenya Zoonotic Disease Unit, Ministry of Health and Ministry of Agriculture, Livestock and Fisheries, Nairobi, Kenya; 5 Animal and Human Health Program, International Livestock Research Institute, Nairobi, Kenya; 6 Division of Global Health Protection, United States’ Centers for Disease Control and Prevention, Nairobi, Kenya; US Army Medical Research Institute of Infectious Diseases, UNITED STATES

## Abstract

**Background:**

In mid-2015, the United States’ Pandemic Prediction and Forecasting Science and Technical Working Group of the National Science and Technology Council, Food and Agriculture Organization Emergency Prevention Systems, and Kenya Meteorological Department issued an alert predicting a high possibility of El-Niño rainfall and Rift Valley Fever (RVF) epidemic in Eastern Africa.

**Methodology/Principal findings:**

In response to the alert, the Kenya Directorate of Veterinary Services (KDVS) carried out an enhanced syndromic surveillance system between November 2015 and February 2016, targeting 22 RVF high-risk counties in the country as identified previously through risk mapping. The surveillance collected data on RVF-associated syndromes in cattle, sheep, goats, and camels from >1100 farmers through 66 surveillance officers. During the 14-week surveillance period, the KDVS received 10,958 reports from participating farmers and surveillance officers, of which 362 (3.3%) had at least one syndrome. The reported syndromes included 196 (54.1%) deaths in young livestock, 133 (36.7%) abortions, and 33 (9.1%) hemorrhagic diseases, with most occurring in November and December, the period of heaviest rainfall. Of the 69 herds that met the suspect RVF herd definition (abortion in flooded area), 24 (34.8%) were defined as probable (abortions, mortalities in the young ones, and/or hemorrhagic signs) but none were confirmed.

**Conclusion/Significance:**

This surveillance activity served as an early warning system that could detect RVF disease in animals before spillover to humans. It was also an excellent pilot for designing and implementing syndromic surveillance in animals in the country, which is now being rolled out using a mobile phone-based data reporting technology as part of the global health security system.

## Author summary

Occurrence of Rift Valley Fever (RVF) outbreak is associated with heavy El-Niño rainfall. In July 2015, an alert on the likelihood of El-Niño rainfall and RVF outbreak in Eastern Africa region was issued by the United States, Food and Agriculture Organization, and Kenya Meteorological Department. In response to the alert, the Kenya Directorate of Veterinary Services (KDVS) carried out an enhanced syndromic surveillance system between November 2015 and February 2016 in the 22 counties that had previously been identified as RVF high-risk counties. The surveillance system collected data on RVF-associated syndromes and risk factors in cattle, sheep, goats and camels from more than 1100 farmers. Of the 10,958 field reports submitted, 45 were consistent with suspect RVF disease and 24 of these identified as probable RVF, triggering an immediate response. Whereas investigations of the suspect cases and laboratory testing did not confirm RVF cases, the surveillance system served as an excellent early warning system that could detect disease in animal before spillover to humans.

## Introduction

Rift Valley Fever (RVF) is a mosquito borne viral zoonoses that primarily affects cattle, goats, sheep, and camels in Africa and the Arabian Peninsula [1–3]. Humans become infected through close contact with blood and organs of infected animals or through bites from an infected mosquito [4]. Epidemics of RVF are a major global health security threat due to the high morbidity and mortality in humans, and the economic impact associated with loss of livestock and ban in international trade. The World Organization for Animal Health (OIE) identifies RVF as an important transboundary and notifiable disease because of its potential for rapidly spreading across international borders, resulting in devastating economic effects through losses in the international trade of animals and animal products [5–8]. RVF epidemics are characterized by massive livestock abortions and death, resulting in high economic losses associated with animal quarantines and trade restrictions [9]. For example, the economic losses resulting from the 2006–2007 RVF epidemic in Kenya were estimated at US $32 million [7]. In humans, over 80% of RVF virus-infected humans are either asymptomatic or have a mild influenza-like illness; however, high morbidity and mortality has been reported in some outbreaks [4,10–13]. A 1977 RVF epidemic in Egypt resulted in an estimated 200,000 human cases and 600 deaths whereas the RVF outbreak in East Africa (Kenya, Somalia, Tanzania) during 1997–1998 resulted in over 100,000 cases and over 450 deaths in Kenya [10,12–14]. A RVF epidemic in Saudi Arabia and Yemen in 2002 resulted in an estimated 4000 human cases and over 200 deaths [2,3].

Globally, livestock RVF epidemics have been most frequently reported in Eastern Africa, occurring every 4 to 10 years and closely linked with periods of heavy rainfall that occur during the warm phase of the El Niño/Southern Oscillation phenomenon [15]. Predictions of RVF epidemics in the region can be given up to 5 months in advance, based on ecological parameters and satellite imagery [16]. In mid-2015, the United States’ Pandemic Prediction and Forecasting Science and Technical Working Group of the National Science and technology Council, Food and Agriculture Organization Emergency Prevention Systems, and Kenya Meteorological Department all issued alerts predicting a high possibility of El-Niño rainfall and RVF outbreaks in Eastern Africa [17,18]. In response to the alert, the Kenya Directorate of Veterinary Services (KDVS) in the Ministry of Agriculture, Livestock and Fisheries pilot tested an enhanced surveillance system between November 2015 and February 2016 in 22 RVF high-risk counties [19]. In Kenya, as in many resources-limited countries, the routine livestock surveillance is passive where public and private animal health officers must wait for farmers to report animal illness before responding. The aim of the enhanced surveillance reported here was to collect near real-time data on syndromes and risk factors associated with RVF to enhance early detection of the disease in livestock before spill over to humans. We describe how the surveillance was conducted, results of the surveillance, and recommend next steps towards establishing a national syndromic surveillance system in livestock and wildlife populations in Kenya.

## Materials and methods

### Selection of high-risk counties

**Fig 1:**
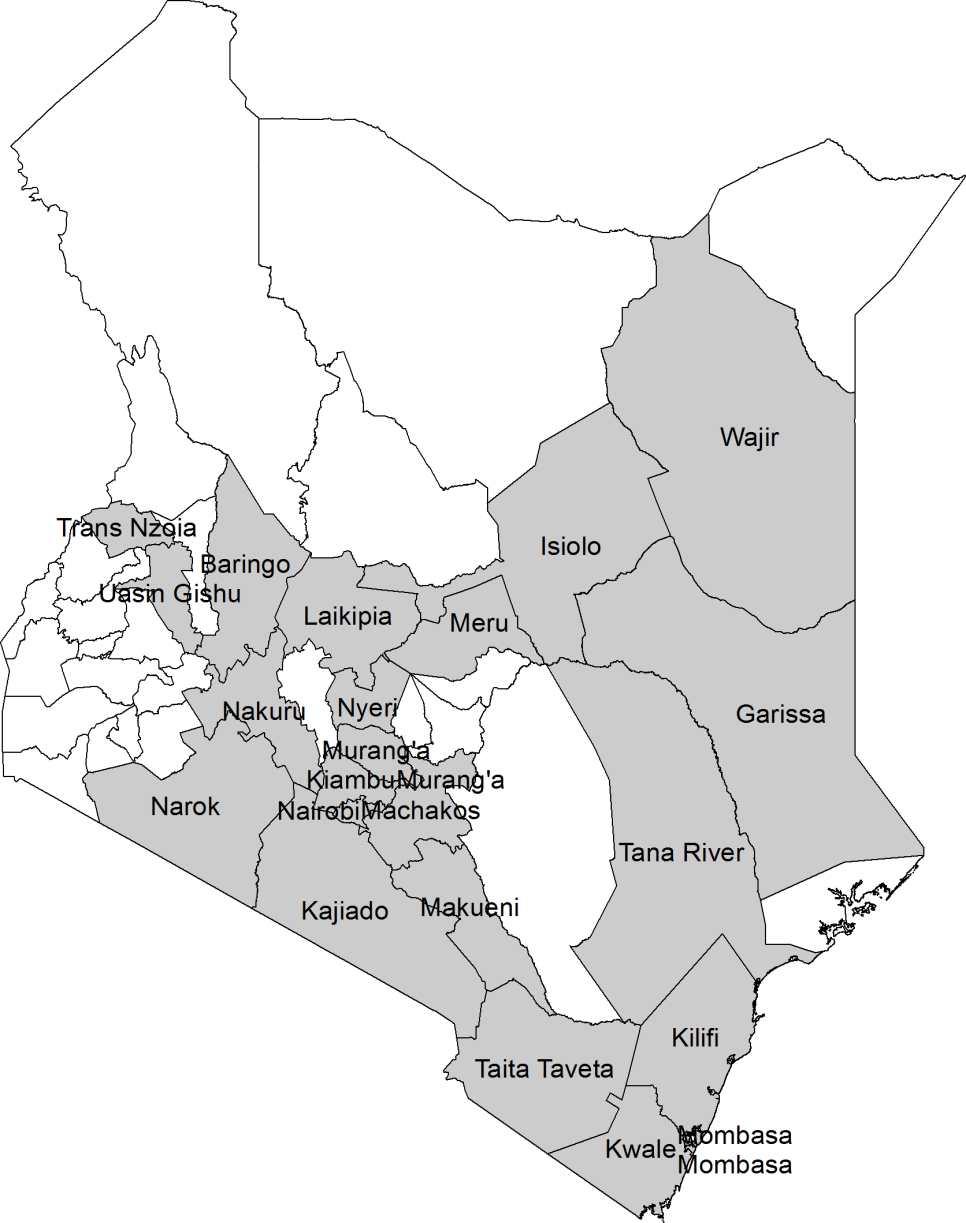
Map of Kenya showing selected Rift Valley Fever (RVF) high-risk counties in grey. A high-risk county was based on ecological and climatic factors associated with previous RVF outbreaks in Kenya, as defined by Munyua et al. [19]. The high-risk counties were selected for participation in the enhanced surveillance for RVF.

To increase the chances of early detection of RVF disease in livestock (cattle, sheep, goats, and camels), an enhanced surveillance system was implemented over a 14-week between November 2015 and February 2016 in the 22 counties at a high-risk of RVF outbreak (out of the 47 counties in Kenya). The 22 RVF high-risk counties shown in Fig 1 had previously been identified through the RVF risk map for the country [19]. In each of the high-risk counties, we selected three sub-counties with the greater risk of the epidemic for the enhanced surveillance. The criteria used to select the sub-counties included the number of susceptible livestock, areas prone to flooding, and history of RVF outbreaks. For the 25 counties that were not at RVF high-risk and therefore not targeted with the enhanced RVF surveillance, routine RVF surveillance was maintained by KDVS.

### RVF reporting system

**Fig 2:**
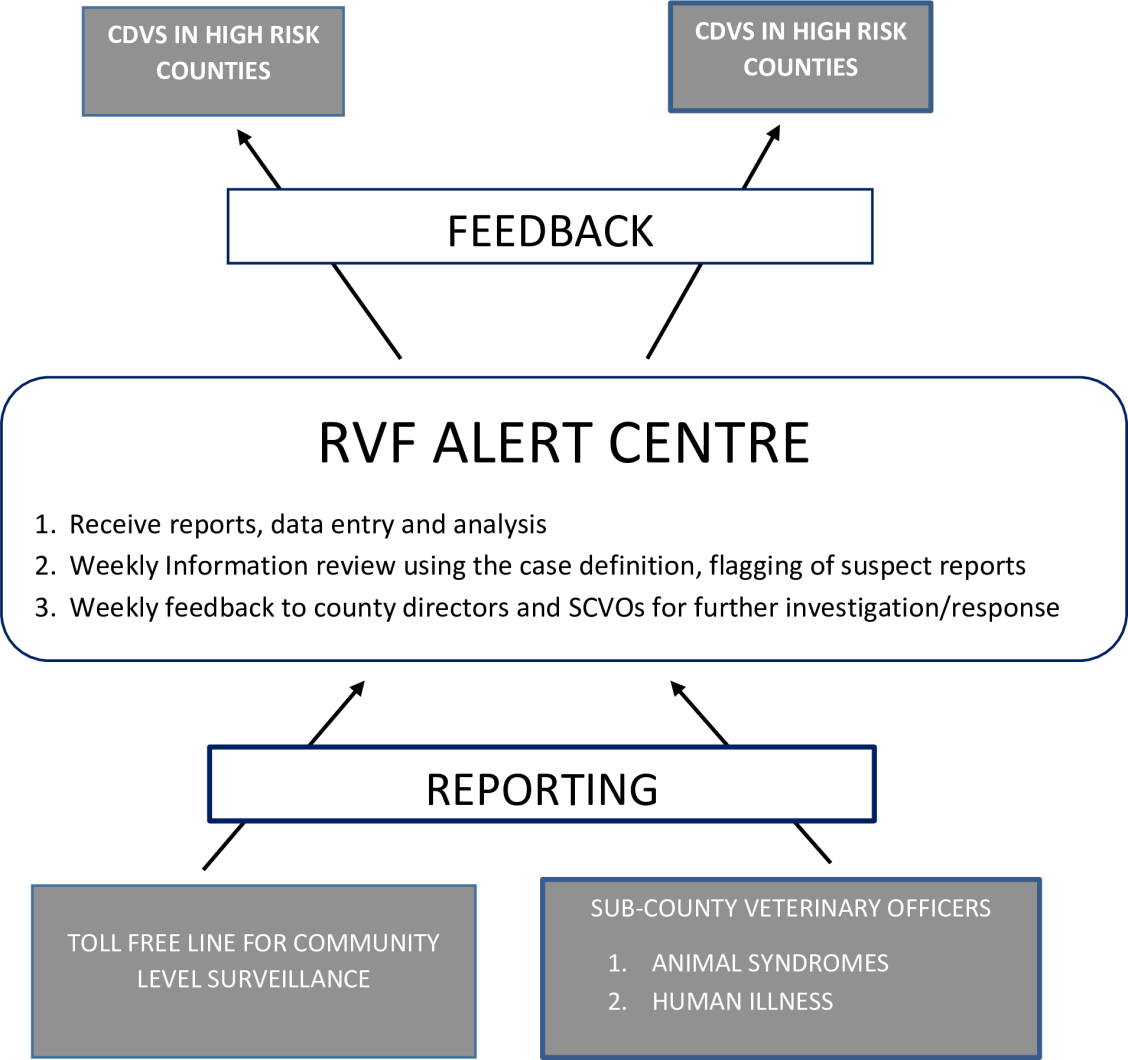
Illustration of RVF surveillance system conducted in Kenya between November 2015 and February 2016. CDVS = County Director of Veterinary Services, RVF = Rift Valley Fever, SCVO = Sub-County Veterinary Officers.

The surveillance system consisted of an RVF Alert Center at the KDVS headquarters to receive, compile and report the surveillance data from the ub-county veterinary officers (SCVOs) who carried out the surveillance at sub-county level, and the livestock farmers who provided the information to the SCVO (Fig 2). The SCVO in each sub-county was responsible for reporting cases of suspected RVF in livestock from the selected farms in their area, using a data collection tool developed for RVF reporting. Each SCVO identified 20 livestock owning farmers evenly spread across the sub-county, and whom they interviewed weekly by telephone to determine whether there were suspected RVF cases in cattle, sheep, goats, camels on their farms or neighboring farms, and any suspect RVF human cases. Weekly, the SCVO collected animal demographic data (farm location, animal numbers and species), RVF risk factors (livestock production system, vaccination status, weather, and vector information), and RVF associated syndromes (abortion, hemorrhagic disease, mortalities and human illness). The SCVOs sent reports every Friday to the RVF Alert Centre via email (Fig 2). The 20 farmers in each in high-risk counties were also trained to use the toll-free number and report directly to the RVF Alert Center. Located at Veterinary Epidemiology and Economics Unit (VEEU) at the KDVS headquarters, the RVF Alert Centre was managed by two veterinary epidemiologists each reachable round the clock through a toll-free numbers.

Reports to the RVF Alert Center were reviewed daily and the County Director of Veterinary Services in area informed within 24 hours, who in turn carried out further investigation and appropriate response. Suspected RVF illness in humans were reported to the County Director of Health in the area, and the Disease Surveillance and Response Unit of the Kenya Ministry of Health headquarters for investigation.

### Case definitions and response

A suspected RVF herd was defined as a herd reporting abortion in any of the livestock in the herd in an area experiencing heavy rainfall and flooding. A probable RVF herd was defined as a herd reporting abortions, mortalities in the young ones, and/or hemorrhagic signs in any of the livestock in the herd in an area experiencing heavy rainfall and flooding. A confirmed RVF herd was defined as a herd where an animal tested positive to RVF by RVF IgM ELISA. Each suspected or probable RVF herd was investigated by the SCVO of the area and reports sent to the RVF Alert Center.

### Sample collection and testing

During the follow-up investigation, the SCVO collected blood samples from suspected or probable herds and shipped them to the Central Veterinary Laboratories (CVL) at Kabete, Nairobi for testing. The presence of anti-RVF immunoglobulin (IgG) and IgM antibodies in sera was determined using the IDVet enzyme linked immunosorbent assay (ELISA) kits according to the manufacturer’s instructions (IDVet Innovative Diagnostic, Grabels, France). For detection of anti-RVF IgG antibodies, ELISA plates were coated with RVF virus recombinant nucleoprotein overnight before washing and adding 50ul of the test serum at 1:10 dilution. A positive and negative control sera were provided in the kit. The plates were incubated for one hour at 37°C, washed, and anti-RVF nucleoprotein peroxidase conjugate added. Following 30 mins incubation, the plates were washed and presence of anti-RVF IgG detected using odometer. For detection of anti-RVF IgM antibodies, anti-bovine, ovine, or caprine (for cattle, sheep and goat sera) IgM polyclonal antibodies were used to coat ELISA plates overnight, washed and test serum added at 1:10 dilution. Plates were incubated for 1 hour at 37°C, washed, and RVF nucleoprotein added and results recorded.

### Rainfall data

Actual rainfall data for the surveillance period (November 2015 to February 2016) were obtained from the Tropical Rainfall Measuring Mission supported by the United States’ National Aeronautics and Space Administration (https://pmm.nasa.gov/precipitation-measurement-missions). The data used were combined microwave-IR-gauge estimates generated from Version 7 Tropical Rainfall Measuring Mission (TRMM) Multi-Satellite Precipitation Analysis algorithm. Rainfall data for November 2015 to February 2016 (files 3B43.20151001.7.nc– 3B43.20160201.7.nc) with a resolution of 0.25° were downloaded and exported into R statistical software [20] for extraction. The extraction used the current Kenya Counties shape file obtained from the Kenya Bureau of Statistics. The extraction function (*extract (rainfall data*, *counties shape file)* is supported by the raster package in the R software.

### Data management and analysis

Data received from the SCVOs and toll-free numbers were entered into a Microsoft access database. Each report was given a unique identification number. Data cleaning involved an independent, process with two-persons checking all data entries to ensure that duplications and errors were removed. Complete data entries were those containing name and contacts of the farm/farmer, location of the farm, size of the herd and number of animals affected per species for each syndrome, humans affected; and associated environmental conditions.

All data were exported as a Microsoft Excel 2010 (Microsoft Corp., Redmond, WA, USA) file for data cleaning which was imported into STATA version 14 (StataCorp, College Station, TX, USA) where data variables were summarized to check for outliers. Suspected and probable RVF herd reports were flagged from these data, and descriptive analyses were performed to generate weekly plots of the RVF cases, and compared with the reported weather conditions and actual rainfall data. Correlations and associations between data variables were assessed by the value of Pearson’s correlation coefficient and Pearson’s Chi-Squared test of significance. The descriptive and statistical analyses were performed in both STATA and Tableau Desktop 10.0 (Tableau Software, Seattle, WA, USA) and geographic visualization performed in ArcMap 10.3.1 (ESRI, Redlands, CA, USA).

### Ethical approval

This surveillance was part of the routine government of Kenya’s response to the threat of RVF outbreak. Therefore, it did not require ethical approval.

## Results

### Enhanced surveillance system

Between November 2015 and February 2016, 56 of the 66 (84.8%) sub-counties in 22 selected counties participated in the RVF enhanced surveillance system for the entirety of the 14-week period. A total of 1,102 of the targeted 1,120 farmers (98.4%) participated. This resulted in 10,958 reports submitted to the RVF Alert Center that were 100% complete. Each surveillance officer submitted an average of 670 (range 297–898) reports per week. Of these reports, 49.3% were from mixed farm production systems, 19.9% from pastoral, 17.8% from agro-pastoral, 10.3% from zero grazing, 1.5% from group ranches, and 1.2% from commercial ranch farming systems.

### Reported animal syndromes

**Fig 3:**
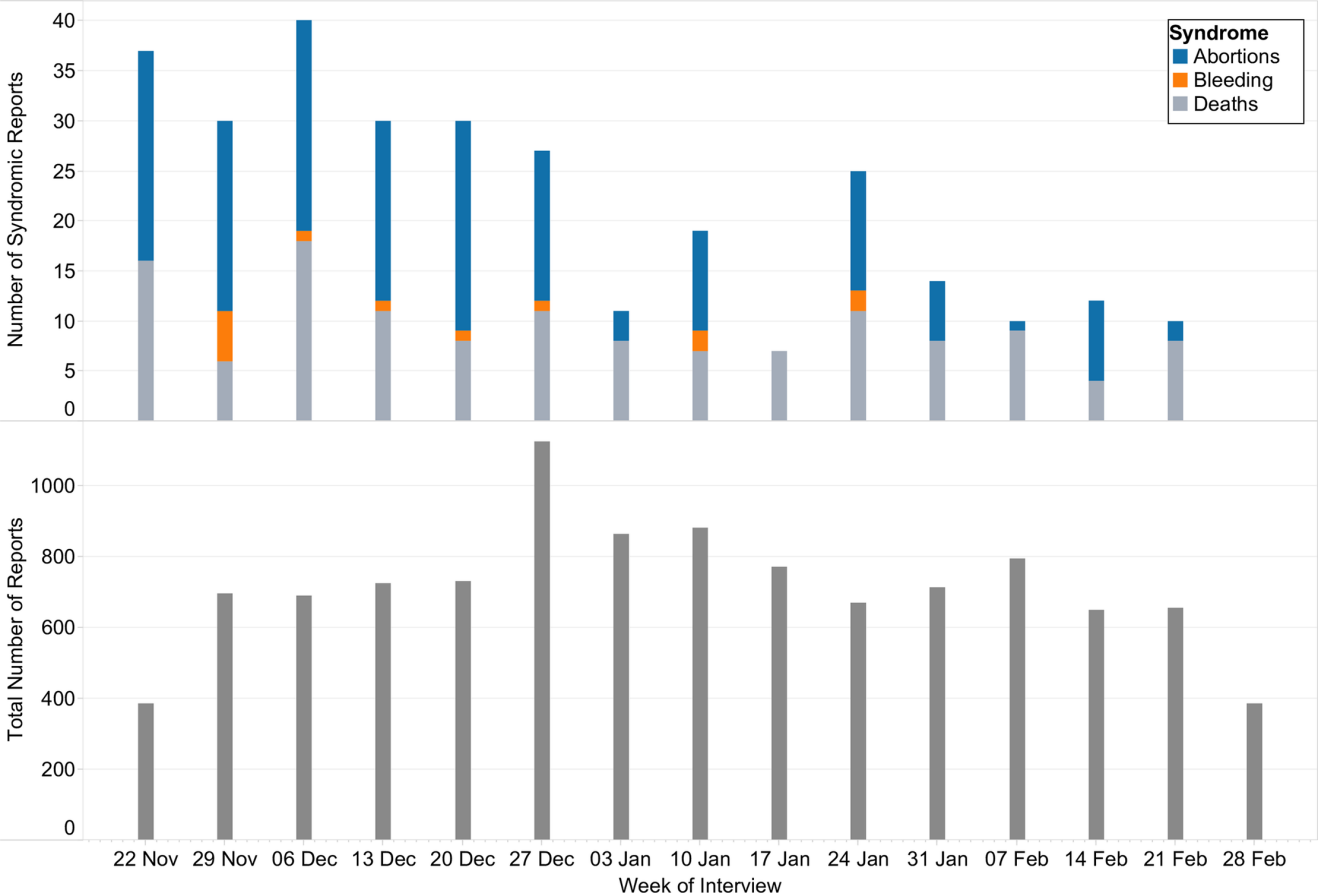
Weekly number of abortions, bleeding, and death syndrome reports (top graph) and number of total reports (bottom graph) submitted to the Rift Valley Fever Alert Centre in Kenya, November 16, 2015 –February 29, 2016. The total number of reports includes both syndromic and healthy reports.

Abortions, bleeding and deaths syndromes were reported in all species (Table 1). A time-series plot of reports submitted during the study period by week is shown in Fig 3. Of the 10,958 syndromic and non-syndromic reports submitted, 362 (3.3%) had at least one syndrome observed within livestock. Of all reported syndromes, 196 (54.1%) were deaths in young livestock, 133 (36.7%) abortions, and 33 (9.1%) hemorrhagic diseases. Abortion and hemorrhagic bleeding were reported more frequently in the first two months (November and December), whereas death in young animals was reported consistently throughout the surveillance period (Fig 3).

**Table 1:** Number of livestock species observed with abortion, bleeding, or death*.

	Syndromes					
	Abortions		Bleeding		Death	
Species	Number sick	Total at risk	Number sick	Total at risk	Number sick	Total at risk
Cattle	489	80,789	16	207	304	56,931
Goats	262	241,292	31	153	1,288	241,132
Sheep	504	286,376	109	674	1,344	216,764
Camels	15	117	18	21	78	27,592

*The totals at risk are different because the syndromes were reported form different herds.

**Fig 4:**
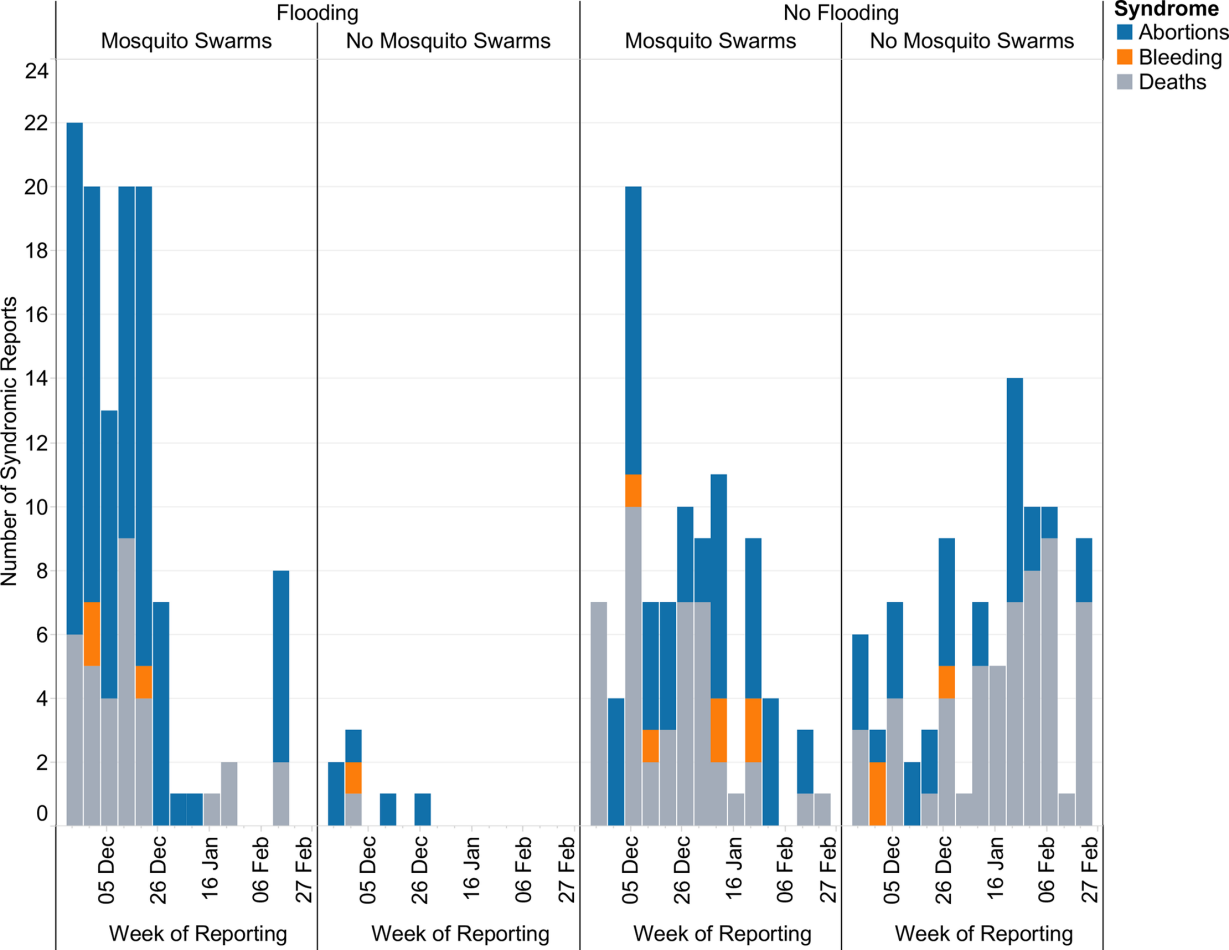
Time-series plots of the weekly reporting for each syndrome by whether flooding and/or mosquito swarms were observed by farmers in their area.

**Fig 5:**
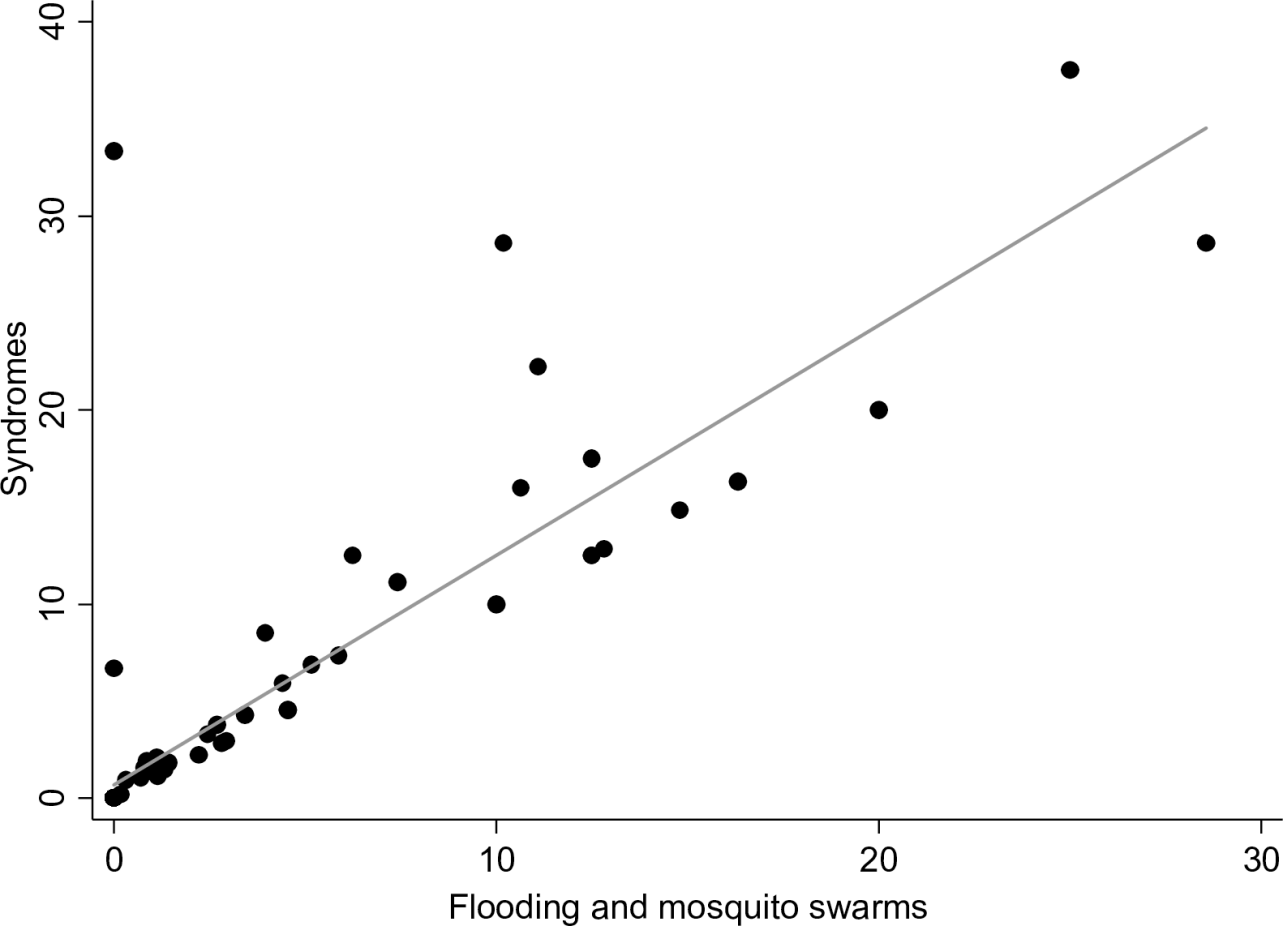
Scatterplot and linear prediction of the correlation between the reporting of any syndrome (abortion, bleeding, or death) and observing flooding and mosquito swarms. These variables showed high correlation, based on Pearson’s correlation coefficient (r > 0.87, p<0.001).

To evaluate the relationship between the reported syndromes and rainfall, we correlated the time-series plot of weekly reports of syndromes with reports of flooding and mosquito swarms (Fig 4). Across syndromes, 211 out of 362 (58.3%) were reported when no flooding was observed. In contrast, more syndromes (69.3%) were reported when mosquito swarms were observed. The reporting across all syndromes with observations of flooding and mosquito swarms were similar with high correlation (Pearson’s correlation coefficient, r> 0.87 and p<0.001). Fig 5 shows the correlation between these variables).

### RVF Suspected and probable herds and cases in livestock

**Fig 6:**
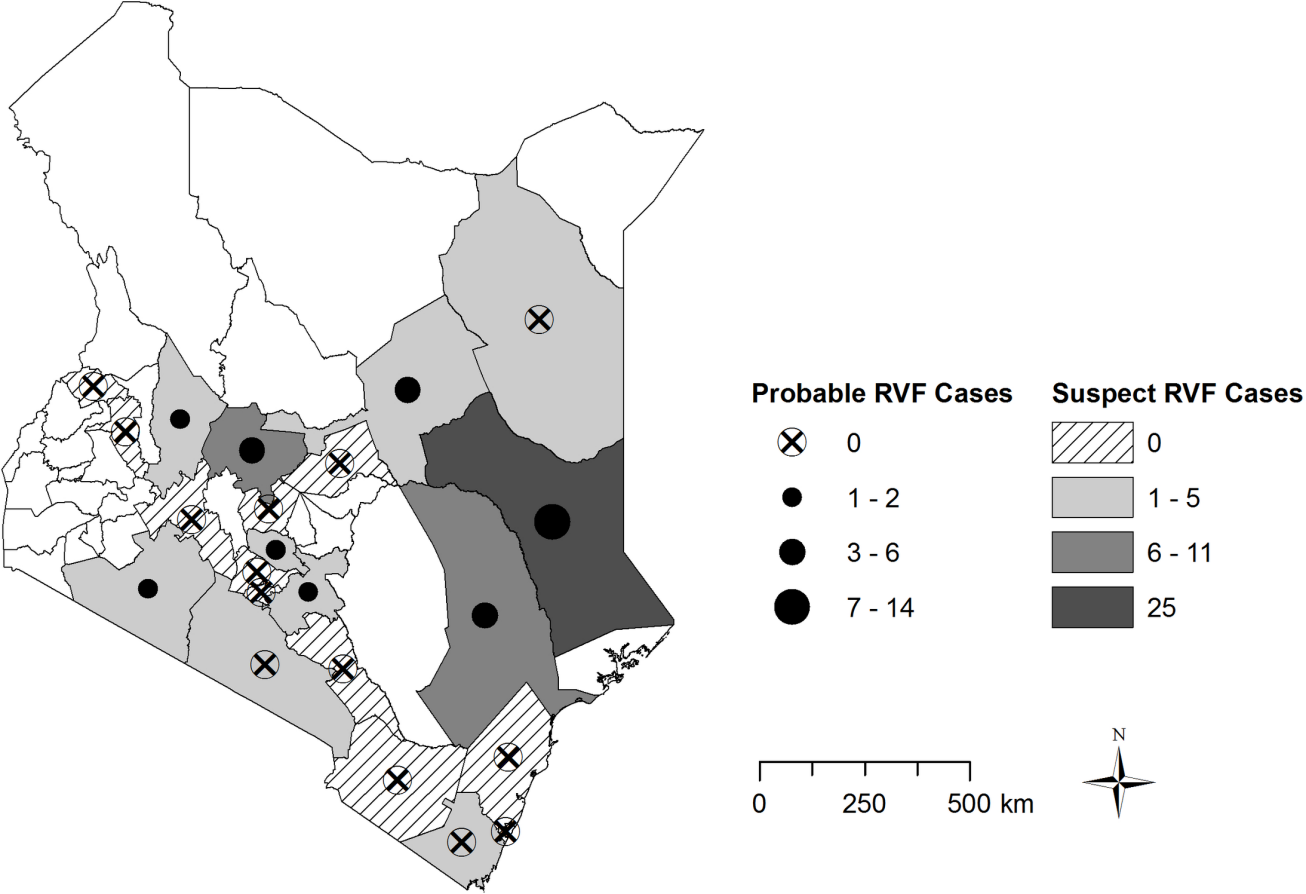
Geographic distribution of the number of Rift Valley Fever suspect and probable herds in Kenya between November 16, 2015 and February 29, 2016. A suspected RVF herd was defined as a livestock herd reporting abortion in an area experiencing heavy rainfall and flooding. A probable RVF herd was defined as a suspect RVF case that also reported deaths in young livestock and/or hemorrhagic signs.

**Fig 7:**
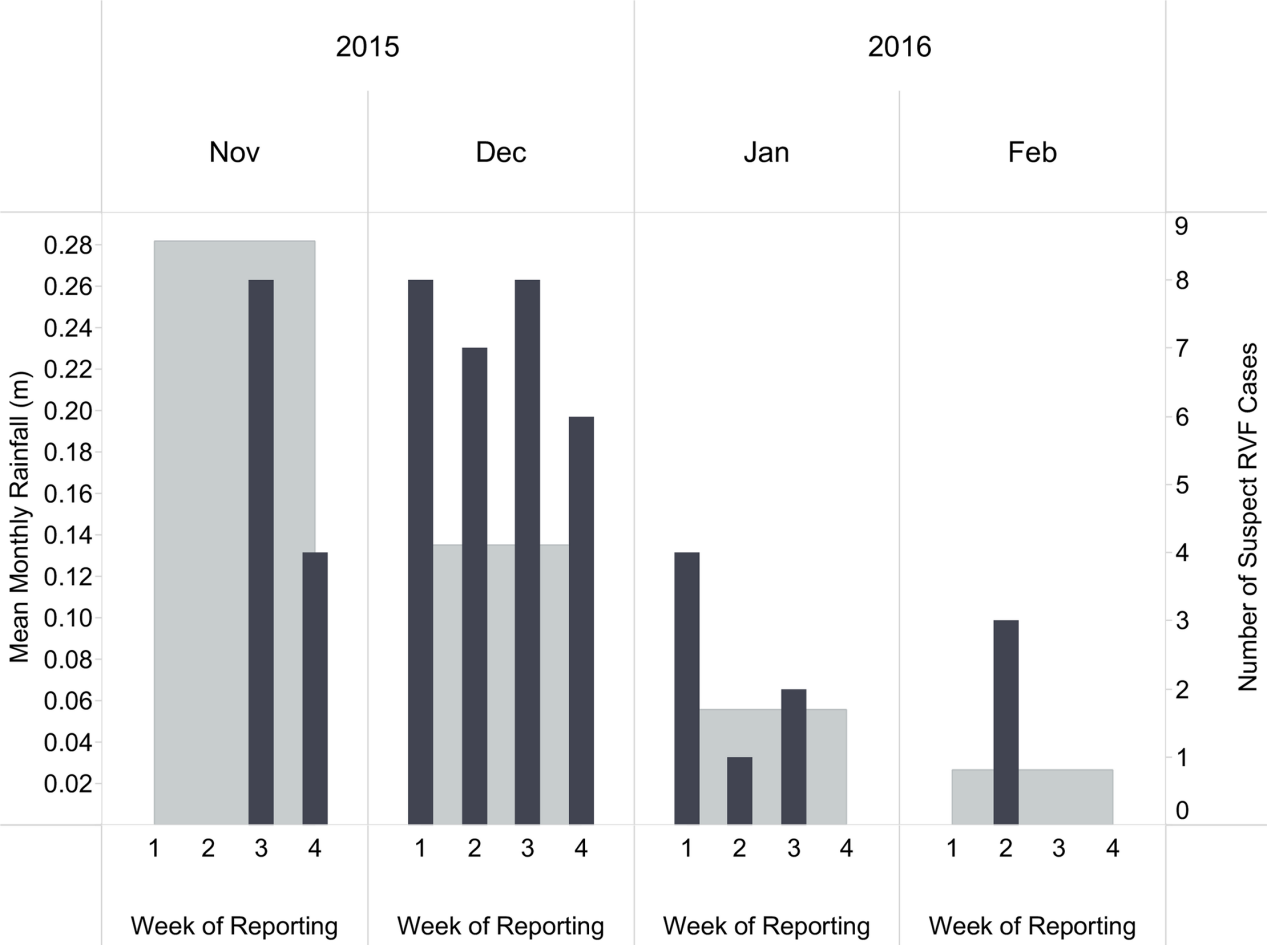
Mean monthly actual rainfall (light shaded bars and left x-axis) recorded in the participating counties and number of suspect and probable Rift Valley Fever (RVF) herds (dark shaded bars and right x-axis) reported in Kenya, November 2015 –February 2016.

A total of 69 (19.1%) suspected RVF cases (abortion in flooded area) from 45 farmers in 10 counties were identified. Of these 24 (6.6%) cases from 18 farmers in 7 counties met the definition for a probable RVF herd. Fig 6 presents the geographic distribution of RVF suspect and probable herds in the study region. Fig 7 plots the suspect RVF herds and actual rainfall over study period. The majority (45 of 69) of suspect RVF herds were reported in November and December 2015, whereas three probable RVF herds were reported in both January and February 2016. Although the mean monthly actual rainfall was lower than the amount typically observed each year during the same months and counties during this period, more rain occurred during November and December and this was highly associated with increased reporting of suspect RVF herds (Pearson’s Chi-Squared, *χ*^*2*^ = 72.9, p<0.001). Of the total reports submitted (10,958), only 27.0% reported having livestock vaccinated for RVF within the previous three months.

Specimens were collected from animals in 17 of the 24 RVF probable herds. Goats from two herds tested positive to RVF IgG antibodies but they were negative on RVF IgM ELISA (Table 2). Samples from the other herds were negative for both IgG and IgM antibodies.

**Table 2:** Rift Valley Fever IgG ELISA test results by species and county of origin, November 2015 to February 2016, Kenya.

Date	County	Species	No. of samples tested	Number positive*
3/11/2015	Kiambu	Bovine	8	0
4/11/2015	Nyeri	Caprine	13	0
20/11/2015	Machakos	Ovine	102	0
14/12/2015	Taita Taveta	Caprine	77	0
14/12/2015	Taita Taveta	Ovine	22	0
14/12/2015	Mombasa	Bovine	2	0
15/12/2015	Garissa	Caprine	10	3
15/12/2015	Garissa	Ovine	5	0
24/12/2015	Garissa	Ovine	8	0
24/12/2015	Garissa	Caprine	30	0
5/2/2016	Nakuru	Ovine	112	0
15/1/2016	Makueni	Caprine	1	0
11/2/2016	Isiolo	Ovine	5	0
11/2/2016	Isiolo	Caprine	13	1
17/2/2016	Trans-nzoia	Ovine	78	0
29/2/2016	Makueni	Bovine	9	0
29/2/2016	Makueni	Caprine	23	0

*Test results are for anti-RVF IgG antibodies. All IgG positive samples were also tested for anti-RVF IgM but none were positive.

## Discussion

Routine livestock surveillance in Kenya is primarily passive, with public and private veterinarians waiting for farmers to report animal illness before responding and reporting. The enhanced surveillance for RVF reported here provided animal RVF disease data that served as an effective early warning for a major outbreak, giving a chance to prevent spillover to humans. The pilot created a model communication network for emergency reporting of animal health status between farmers, county government surveillance officers, and the national government. While the pilot focused on a select number of farmers, it demonstrated the willingness of farmers to participate, which is vital for the success of any national syndromic surveillance system [21]. Although the predicted heavy El Niño rainfall that is associated with RVF outbreaks was not received in the East Africa region, the occurrence pattern of syndromes and RVF herds showed a positive correlation with rainfall and flooding. Overall, the number of reports of RVF-associated syndromes, in particular abortions and hemorrhagic disease were high in the months that reported the highest rainfall (Fig 3). A similar trend was observed with suspected and probable RVF herds (Fig 6). These data resulted in increased awareness among farmers, and animal and human health officers in these areas, thus increasing the chance of detecting RVF cases.

The surveillance had a number of limitations that will be important to address for any future syndromic surveillance efforts in Kenya. Since this was for selected regions, the surveillance and resulting data collected were not representative of the targeted animal populations of interest. While it would then be possible that RVF cases could have occurred and not been detected by this system, it was expected that any other outbreaks would have been reported through regular reporting channels set by the KDVS. Another limitation of this work was that the surveillance officers submitted their reports on a weekly basis, affecting the timeliness of data collection. Furthermore, the data received at the RVF Alert Center had to be manually transferred to another database for analysis, a step that introduced possible additional human error and delays in data analysis. Leveraging current technologies for both data collection (e.g., mobile phones) and data integration/analysis that allow for near real-time reporting of animal health will be required in order for future syndromic surveillance efforts to successfully meet their intended purpose of early detection of disease events. Another limitation is that the laboratory results may not have been representative of disease status of the herd, with a possibility that IgM positivity in the suspected or probable herds was missed. This is because the method of collecting and testing of samples from suspected and probable herds was neither random nor did it target animals with the clinical signs.

There were no RVF outbreaks confirmed during the surveillance period, most likely because the predicted El Niño rainfall was not received. However, it is important to note that 27% of the farmers reported having vaccinated their livestock against RVF within the previous three months, and surveys in these RVF high-risk regions have typicaly reported >10% seropositivity in livestock, and up to 20% seropositivity in humans [22]. Given that occurrence of RVF epidemics seems to require low herd immunity, this level of immunity may have also have reduced the risk of RVF outbreak in the country.

This RVF enhanced surveillance pilot demonstrated the capacity and need for establishing a national syndromic surveillance system in livestock in Kenya. Such a system would need to be synergistic with other surveillance systems in the country so as not to overburden data providers. The fact that both the KDVS and Kenya Wildlife Services do not have established national disease surveillance systems is an advantage as it enables the designing of a system that works in both livestock and wildlife. Similarly, guidelines would need to be established between the responsible animal and public health government agencies so as to ensure the infrastructure is in place to handle the additional information, and to determine appropriate responses to potential disease events that are effective and do not overwhelm their resources [23]. An ideal surveillance system should also implement data collection standards and be expanded to include a comprehensive set of clearly defined disease syndromes so as to have the capability to detect transboundary, emerging, and zoonotic disease events. Finally, the system should allow regular and near real-time feedback of the collected data to surveillance officers so as to enhance situational awareness and support the sustainability of the overall system. By leveraging current technologies such as mobile phones that are gaining usage globally for syndromic surveillance, most of the aforementioned successes can be enhanced, and the limitations from this RVF enhanced surveillance can be addressed.

## Conclusions

This surveillance demonstrated the need to establish a national syndromic surveillance system in livestock and wildlife in Kenya. Further, the interaction between humans, animals, and the environment reinforces the concept of syndromic surveillance within the One Health concept [24]. The RVF enhanced surveillance served as an important first step toward designing and implementing an animal syndromic surveillance system in Kenya.

As follow-up to these efforts, the United States’ Centers for Disease Control and Prevention (CDC) is currently funding work to develop and deploy syndromic surveillance system in domestic animals and wild animals in Kenya, using a mobile and data integrations/analysis technologies customized for the country, referred to as the Kenya Animal Biosurveillance System (KABS). The KABS is capable of integrated analysis of animal and public health data using algorithms defined by veterinary officers within the Kenya government. The KABS technology will allow data providers and government animal health officials to quickly detect and report the animal health status in domestic animals and wildlife populations across different geographical areas and provide early warning information from validated sources signaling activity to assist in decision-making and response during a disease event. Furthermore, KABS will be the first instance of implementing routine surveillance in Kenya wildlife populations. Once fully developed, KABS will be a low cost, easy to implement surveillance technology solution that can be customized and adapted to other country’s needs and requirements for supporting human and animal health.
